# Fluorescence tracking demonstrates T cell recirculation is transiently impaired by radiation therapy to the tumor

**DOI:** 10.1038/s41598-024-62871-w

**Published:** 2024-05-24

**Authors:** Gwen Kramer, Tiffany Blair, Shelly Bambina, Aanchal Preet Kaur, Alejandro Alice, Jason Baird, David Friedman, Alexa K. Dowdell, Michio Tomura, Clemens Grassberger, Brian D. Piening, Marka R. Crittenden, Michael J. Gough

**Affiliations:** 1 Earle A. Chiles Research Institute, Robert W. Franz Cancer Center, Providence Portland Medical Center, Portland, OR 97213 USA; 2 Laboratory of Immunology, Faculty of Pharmacy, Osaka Ohtani University, Tondabayashi, Osaka 584-8540 Japan; 3 Department of Radiation Oncology, University of Washington, Fred Hutch Cancer Center, Seattle, WA USA; 4 The Oregon Clinic, Portland, OR 97213 USA

**Keywords:** Immunosurveillance, Radiotherapy

## Abstract

T cells recirculate through tissues and lymphatic organs to scan for their cognate antigen. Radiation therapy provides site-specific cytotoxicity to kill cancer cells but also has the potential to eliminate the tumor-specific T cells in field. To dynamically study the effect of radiation on CD8 T cell recirculation, we used the Kaede mouse model to photoconvert tumor-infiltrating cells and monitor their movement out of the field of radiation. We demonstrate that radiation results in loss of CD8 T cell recirculation from the tumor to the lymph node and to distant sites. Using scRNASeq, we see decreased proliferating CD8 T cells in the tumor following radiation therapy resulting in a proportional enrichment in exhausted phenotypes. By contrast, 5 days following radiation increased recirculation of T cells from the tumor to the tumor draining lymph node corresponds with increased immunosurveillance of the treated tumor. These data demonstrate that tumor radiation therapy transiently impairs systemic T cell recirculation from the treatment site to the draining lymph node and distant untreated tumors. This may inform timing therapies to improve systemic T cell-mediated tumor immunity.

## Introduction

T cells infiltrating the tumor are within the treatment field of radiation therapy and are readily killed by radiation. Treatment has the potential to disproportionately negatively impact tumor-specific T cell populations^1^. Nevertheless, a range of tumor models rely on T cells for optimum control of tumors following radiation therapy^2^. One explanation of this data is that some tumor-specific T cells evade the treatment field through recirculation. Recirculation describes the mechanism by which lymphocytes pass through peripheral sites to scan for cognate antigen, returning via the lymphatics back into the circulation^3^. The effect of radiation therapy on T cell recirculation dynamics has not been previously characterized.

Snapshot analyses of tumor infiltration after radiation suggests differing radiosensitivity of T cell subsets, for example T regulatory cells may be less radiosensitive^4,5^; however, the impact of differential repopulation following treatment^6–8^ or altered local proliferation following treatment^9^ will contribute to apparent radiosensitivity. Recent data demonstrates that radiation therapy of the tumor-draining lymph node (TdLN) negatively impacts the immune response to radiation therapy^10,11^ likely by killing critical tumor-specific T cells in the TdLN. Radiation strategies that avoid the TdLN or circulating immune cells have been proposed as an approach to avoid depletion of tumor antigen specific T cells^12,13^; however, if the dominant population of tumor-specific T cells is still killed upstream in the tumor environment, radiation-mediated changes to recirculation may directly impact the utility of the TdLN as a site to regulate anti-tumor immunity.

To address the impact of radiation of the tumor on T cell recirculation through the TdLN, we used the Kaede photoconvertible mice. Kaede mice express the Kaede-Green fluorescent protein that can be converted into the Kaede-Red fluorescent protein upon exposure to violet light^14^. This approach has allowed us to perform site-specific fluorescence tagging of tumor infiltrating host dendritic cells and identify these cells when they traffic to the TdLN^15^. Using tumor-specific photoconversion, we demonstrate that radiation therapy results in a loss of CD8 T cell recirculation from the tumor to the TdLN at early time points following radiation. Using a panel of murine cell lines, we demonstrate that the number of cells recirculating to the TdLN is linked to the number of T cells in the tumor and the decrease following radiation is linked to cytotoxicity in the tumor. Using scRNASeq, we demonstrate loss of a proliferative population in the tumor immediately following radiation therapy. The loss of recirculating T cells following radiation therapy in turn negatively impacts T cell immune surveillance of distant unirradiated tumors. This decrease is transient, at later time-points recirculation to the TdLN of irradiated tumors is increased compared to unirradiated controls. Together, these data demonstrate a novel dynamic understanding of T cell movement following radiation therapy, are crucial to design radiotherapy regimen minimize the adverse impact of radiation on tumor-directed immunity, and may inform timing of interventions that interact with these recirculating T cells.

## Results

### Kaede photoconversion model to track recirculation

**Figure 1:**
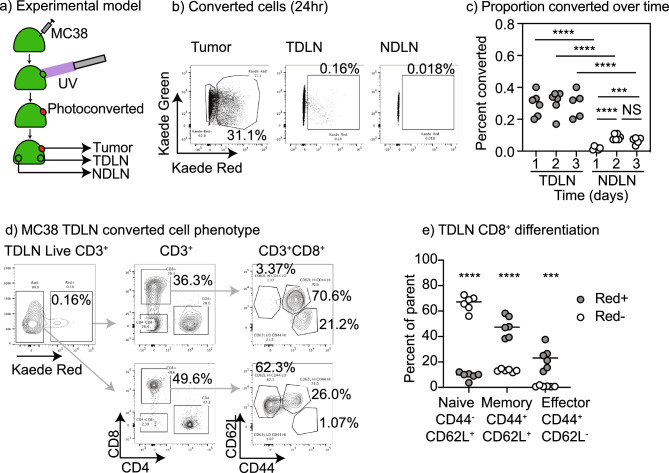
Identification of tumor origin recirculating CD8 T cells in lymph nodes. (**a**) Kaede mice were implanted with MC38 tumors and at d14 tumors were selectively photoconverted with UV light. Tumors, TdLN and NdLN were harvested over time. (**b**) Identification of photoconverted Kaede Red cells in the tumor, TdLN, and NdLN 24 h following photoconversion of the tumor. (**c**) Percent of TdLN and NdLN cells that are photoconverted over time. (**d**) Phenotype of photoconverted CD3^+^ cells in the TdLN at 24 h. (**e**) Quantification of the proportion of Photoconverted (Red^+^) versus non-photoconverted (Red^−^) CD8 T cells that are naïve, memory, or effector differentiated in the TdLN. Key. *NS* not significant; ***p < 0.001; ****p < 0.0001.

Kaede mice express a green fluorescent protein that can be photoconverted to a red fluorescence by exposure to UV light^14^. Mice were implanted with MC38 colorectal carcinoma cells to form tumors, and established tumors were treated with UV light while shielding all other sites to result in selective photoconversion of cells in the tumor (Fig. 1a). Analysis of the tumor draining lymph node (TdLN) and distant non-draining lymph nodes (NdLN) over time demonstrated converted cells were enriched in the TdLN compared to the NdLN (Fig. 1b), confirming prior data^16^. Converted CD8 T cells became more frequent in NdLN of mice at day 2 to 3 after UV exposure (Fig. 1c) suggesting that T cells that originated in the tumor progressively spread throughout the animal. As a natural consequence of the unguided dissemination of lymphocytes via blood circulation the proportion of converted cells in the distant lymph node was an order of magnitude lower than that in the TdLN (Fig. 1c). To better characterize the converted and unconverted cells in the TdLN, cells were separately gated based on photoconversion and further phenotyped (Fig. 1d). Along with CD8 T cells we can identify CD4 T cells that have migrated from the tumor to the TdLN and a significant population of CD8^−^CD4^−^ T cells that have been identified as gamma delta T cells^17^. The converted CD8 T cells in the TdLN were almost exclusively antigen experienced, as shown by expression of CD44, and were a mixture of CD44^+^CD62L^+^ central memory and CD44^+^CD62L^−^ effector/effector memory cells (Fig. 1d). By contrast, the majority of unconverted cells in the TdLN were CD44^−^CD62L^+^ naïve T cells (Fig. 1d,e). The numerical dominance of the unconverted population means that, without the Kaede marker, any gating of T cells in the TdLN is overwhelmingly dominated by cells that did not traffic to the TdLN from the tumor. These data demonstrate that the photoconversion model allows us to identify the T cells in the TdLN that have entered via recirculation through the tumor.

### Radiation effects on T cell recirculation

**Figure 2:**
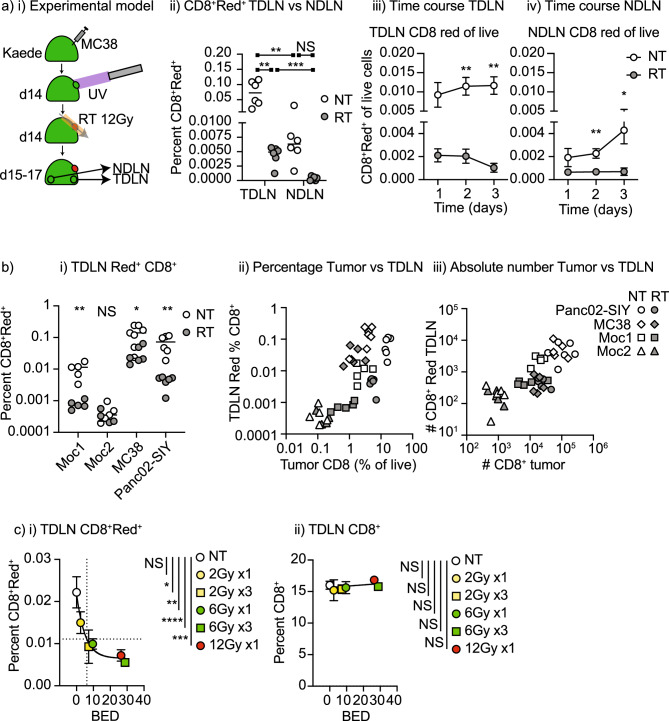
The effect of tumor radiation therapy on recirculating CD8 T cells in lymph nodes. (**a**) (**i**) Kaede mice were implanted with MC38 tumors and at d14 tumors were selectively photoconverted with UV light. Tumors were irradiated with CT-guided radiation to the tumor using a SARRP following photoconversion. Tumors, TdLN and NdLN were harvested over time. (**ii**) Identification of photoconverted Kaede Red cells in the TdLN, and NdLN 24 h following photoconversion in untreated tumors or tumors that are treated with 12 Gy radiation. Percent of (**iii**) TdLN and (**iv**) NdLN cells that are photoconverted over time. (**b**) (**i**) Summary of the proportion of photoconverted CD8 T cells in the TdLN of Moc1, Moc2, MC38, and Panc02-SIY tumors when left untreated or treated with 12 Gy radiation to the tumor on a log scale. (**ii**) Correlation between the photoconverted CD8 T cells in the TdLN and the percent CD8 T cell infiltration of the tumors on a log scale. Open symbols are untreated tumors, closed symbols are tumors treated with 12 Gy radiation. (**iii**) Data from (**ii**) represented as absolute numbers in the TdLN or tumor on a log scale. (**c**) The impact of radiation dose and fractionation on photoconverted CD8 T cells in the TdLN of MC38 tumors. Graphs show (**i**) the percent photoconverted CD8 T cells in the TdLN or (**ii**) the percent unconverted CD8 T cells in the TdLN 1d following the last dose of radiation and photoconversion. The x-axis shows BED_10_ for each dose or dose series. Dotted lines show half maximal photoconverted CD8^+^ T cells in the TdLN and corresponding BED_10_. Key. *NS* not significant; *p < 0.05; **p < 0.01; ***p < 0.001; ****p < 0.0001.

To study the effect of radiation, MC38 tumors in Kaede mice were photoconverted and tumors were treated with 12 Gy radiation therapy via CT guidance with care taken to ensure the TdLN remained outside of the treatment field as previously described^18^. The lymph nodes were analyzed for photoconverted cells over time (Fig. 2ai). The proportion of CD8 T cells among photoconverted cells in the TdLN was significantly decreased following radiation therapy when compared to the TdLN of untreated tumors and this decrease was sustained through 3 days following radiation (Fig. 2aiii,iv). Notably, while the NdLN had significantly fewer converted T cells than the TdLN, radiation therapy also decreased converted T cells in the NdLN (Fig. 2aiv), demonstrating that systemic distribution of T cells originating in the tumor is impaired following radiation therapy. There was no change in the proportion of unconverted T cells in the TdLN or NdLN, and there was no detectable decrease in the overall cellularity of the TdLN (Supplemental Fig. 1), thus changes in recirculation would not have been detectable without the cell tracking Kaede model.

To confirm these data in other models, we similarly studied the Panc02-SIY pancreatic adenocarcinoma model and the Moc1 and Moc2 oral carcinoma models (Fig. 2bi). As with the MC38 tumors, in mice bearing either Panc02-SIY or Moc1 tumors, radiation therapy significantly decreased the proportion of photoconverted CD8 T cells in the TdLN (Fig. 2bi). The Moc2 model exhibited a very small proportion of photoconverted CD8 T cells in the TdLN, and this low proportion did not change following radiation therapy (Fig. 2bi). Since Moc2 tumors are known to have a very limited infiltration of CD8 T cells into the tumor environment^19,20^ and they have a very limited number of photoconverted cells moving from the tumor to the TdLN, we investigated whether the number of photoconverted CD8 cells in the TdLN was directly linked to the number of CD8 T cells in the tumor. By comparing the percentage of CD8 T cells in the tumor to the photoconverted CD8 T cells in the TdLN, we see a direct relationship between these proportions (Fig. 2bii). Radiation treatment resulted in an immediate decrease in the proportion of CD8 T cells infiltrating the tumors (Fig. 2bii, Supplemental Fig. 2) and this was matched by a decrease in photoconverted cells in the TdLN. This proportional data was confirmed by quantitative analysis of the absolute numbers of CD8 T cells and photoconverted CD8 T cells in the tumor and TdLN, showing that the number of photoconverted CD8 T cells in the TdLN was directly proportional to the number of CD8 T cells in the tumor (Fig. 2biii). Again, this relationship remained following radiation therapy. These data suggest that the lack of recirculating CD8 T cells in the TdLN results from their loss in the tumor. The relationship between the proportion of CD8 T cells in the tumor and converted cells in the TdLN were sustained on day 2 and 3 days following photoconversion (Supplementary Fig. 3) suggesting that this is not a temporary correlation. T cell exit from tumors is impacted by antigen and actively regulated by chemokines^21^. To confirm that lymphocyte exit from the tumor and into the draining lymphatics is an active process, we examined the role of exit signals via S1P on T cells expressing S1PR1. Blockade of S1P-S1PR1 signals using FTY720 can cause lymphocyte accumulation in tissues^22^, as well as the more commonly appreciated accumulation of lymphocytes in lymph nodes^23,24^. FTY720 treatment at the time of conversion of Kaede^+^ cells in the tumor did not significantly alter the total number of CD3^+^ T cells in the TdLN 1 day later, but there were significantly fewer converted CD3^+^ T cells and converted CD8^+^ T cells in the TdLN (Supplemental Fig. 4). These data confirm that the exit of T cells from the tumor to the lymph node is dependent on active signals through S1P1R. To understand whether there was any defect in egress of cells through irradiated lymphatics, we examined the trafficking of converted dendritic cells to the TdLN. DC are a more radioresistant cell type than T cells^25,26^, but also use lymphatic transit to migrate to TdLN. Following radiation therapy there was no decrease in the number of converted DC in the TdLN (Supplemental Fig. 5), indicating that the lymphatics remain functional following RT. The exception was the Panc02-SIY model, where we have previously shown a failure of maturation limits DC migration to the TdLN following radiation therapy^15^. These data suggest that the failure of recirculation relates to the radiation-mediated loss of T cells in the treated tumor, resulting in fewer cells that can exit the tumor through the draining lymphatics.

Dose and fractionation of radiation therapy are key issues in clinical treatment and are directly relevant to the impact of radiation on T cells^1^. For this reason, we explored whether lymphocyte loss could be avoided using a lower dose (2 Gy and 6 Gy as single doses) or the impact of repeated daily doses over the three days of study (Fig. 2c, Supplemental Fig. 6). The fractionation series 2 Gy × 3 is directly relevant to conventional fractionation of radiation, and 6 Gy × 3 can address the role of additional fractions while maintaining the equivalent biologically effective dose (BED) as 6 Gy × 3 = 28.8 Gy vs. 12 Gy × 1 = 26 Gy)^27^. Photoconversion took place on the day of the last dose (or the single dose), so that we could synchronize the different groups, and TdLN were analyzed 1 day later. A 2 Gy dose of radiation therapy did not statistically significantly impact recirculating CD8 T cell proportions in the TdLN, but a statistically significant loss of photoconverted CD8 T cells was observed at single doses of 6 Gy and 12 Gy (Supplemental Fig. 6). 6 Gy × 3 also reduces the number of recirculating CD8 T cells but even standard fractionation of 2 Gy × 3 significantly reduces the number of recirculating CD8 T cells in the TdLN (Supplemental Fig. 6). If we plot photoconverted CD8 versus BED, we find a 50% percent reduction of photoconverted CD8 T cells occurs at a BED of 6.1 Gy, which corresponds to a single dose of approximately 4.3 Gy (Fig. 2ci). We observed no loss of total CD8 T cells (converted plus unconverted) in the TdLN at any dose, consistent with the small proportion of TdLN cells that are photoconverted (Fig. 2cii). These data demonstrate that lymphocyte tracking can identify a loss of recirculating lymphocytes even at commonly applied hypofractionated doses of radiation and at standard doses with fractionation.

### Recirculating cells are enriched for tumor-specific effector T cells

**Figure 3:**
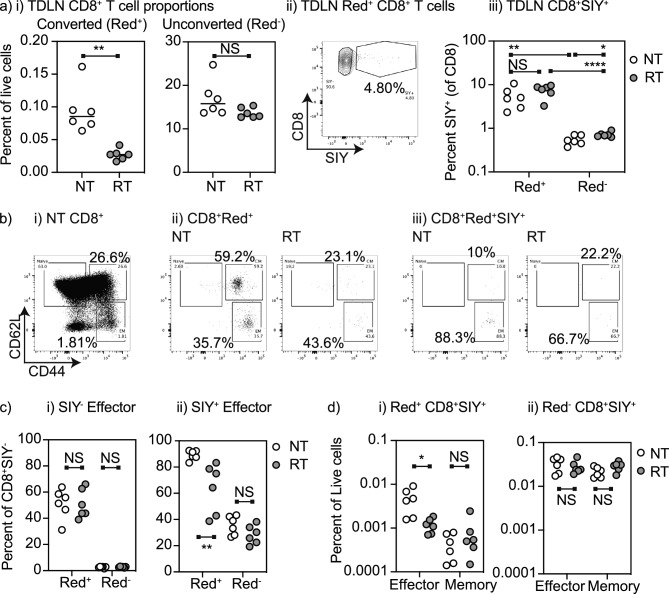
Recirculating tumor antigen specific effector CD8 T cells are impacted by radiation therapy of the tumor. (**a**) Kaede mice were implanted with Panc02-SIY tumors and at d14 tumors were selectively photoconverted with UV light. Tumors were irradiated with 12 Gy CT-guided radiation to the tumor using a SARRP following photoconversion. (**i**) TdLN were harvested and the proportion of photoconverted (Red^+^) and unconverted (Red^−^) CD8 T cells in the TdLN were calculated. (**ii**) Representative gating for the proportion of SIY-specific CD8 T cells in photoconverted (Red^+^) cells, (**iii**) quantification of (**ii**). (**b**) Assessment of antigen experienced (CD44^+^) and effector differentiated (CD62L^−^) cells in TdLN. (**i**) Total CD8^+^ T cells. (**ii**) Photoconverted CD8^+^ T cells in TdLN of untreated or irradiated tumor. (**iii**) Photoconverted SIY-specific CD8^+^ T cells in TdLN of untreated or irradiated tumor. (**c**) Proportion of photoconverted (Red^+^) and unconverted (Red^−^) (**i**) non SIY-specific CD8 T cells or (**ii**) SIY-specific CD8 T cells that are effector (CD44^+^CD62L^−^) differentiated in TdLN. (**d**) Proportion of TdLN cells that are photoconverted (Red^+^) and unconverted (Red^−^) (**i**) non SIY-specific CD8 T cells or (**ii**) SIY-specific CD8 T cells that are effector (CD44^+^CD62L^−^) differentiated in TdLN. Key. *NS* not significant; *p < 0.05; **p < 0.01; ***p < 0.001; ****p < 0.0001.

To understand whether radiation differentially impacts recirculation of non-specific CD8 T cells versus tumor-specific CD8 T cells, we analyzed the TdLN of Panc02-SIY where we can identify SIY-specific CD8 T cells by SIY-MHC-pentamer binding^28^. As before, the proportion of converted CD8 T cells in the TdLN that originated in the tumor was decreased by radiation therapy while the overall number of unconverted CD8 T cells was unchanged (Fig. 3ai). The converted cells in the TdLN were enriched for SIY-specific cells compared to the unconverted cells (Fig. 3aii,iii), consistent with the significant enrichment of tumor-specific CD8 T cells in the upstream tumor. However, radiation did not impact the proportion of converted cells that were SIY-specific (Fig. 3aiii). These data suggest that tumor antigen-specific T cells coming from the tumor are impacted to a similar degree to circulating CD8 T cells of unknown specificity. To better distinguish the phenotypes of tumor antigen-specific CD8 T cells that track to the TdLN, and the effect of radiation on these populations, we identified the distribution of these cells into CD44^+^CD62L^−^ effector and CD44^+^CD62L^+^ memory populations in the TdLN (Fig. 3bi). As observed in mice bearing MC38 tumors, the photoconverted T cells in the TdLN of Panc02-SIY tumors are enriched for memory and effector differentiated T cells (Fig. 3bii). Converted SIY-specific cells in the TdLN were highly enriched for CD44^+^CD62L^−^ effectors (Fig. 3biii), consistent with the fact that the majority of SIY-specific cells in the tumor are also CD44^+^CD62L^−^^29^. CD44^+^CD62L^−^ effector phenotype cells were rare among the unconverted CD8 T cells that did not recognize SIY in the TdLN (Fig. 3ci), indicating that a large proportion of the effector populations in the TdLN either emigrated from the tumor to the TdLN or recognize the immunodominant tumor antigen SIY. Radiation causes a significant decrease in the proportion of converted T cells in the TdLN, and a decrease in the proportion of SIY-specific T cells in the TdLN that are converted (Fig. 3cii). Thus, although the proportion of CD8 T cells that are SIY-specific in the converted cells is unchanged (Fig. 3aiii), the fact that converted cells are enriched for SIY-specific cells and there are fewer converted cells arriving in the TdLN means that there are overall fewer SIY-specific CD8 T cells in the TdLN following radiation. To account for the numerical differences, these data were recalculated as the percentage of SIY-specific T cells of all live cells in the TdLN, demonstrating a selective decrease in converted SIY-specific effector CD8 T cells in the TdLN following radiation therapy with no impact on converted SIY-specific memory CD8 T cells (Fig. 3di) or any non-converted populations (Fig. 3dii). Together, these data indicate that while lymph node trafficking of tumor antigen-specific CD8 T cells is similarly affected by radiation as compared to T cells of unknown specificity, the result is an overall loss of tumor-specific effector T cells in the TdLN following radiation therapy.

### Radiation selectively depletes proliferating CD8 T cells in the tumor

**Figure 4:**
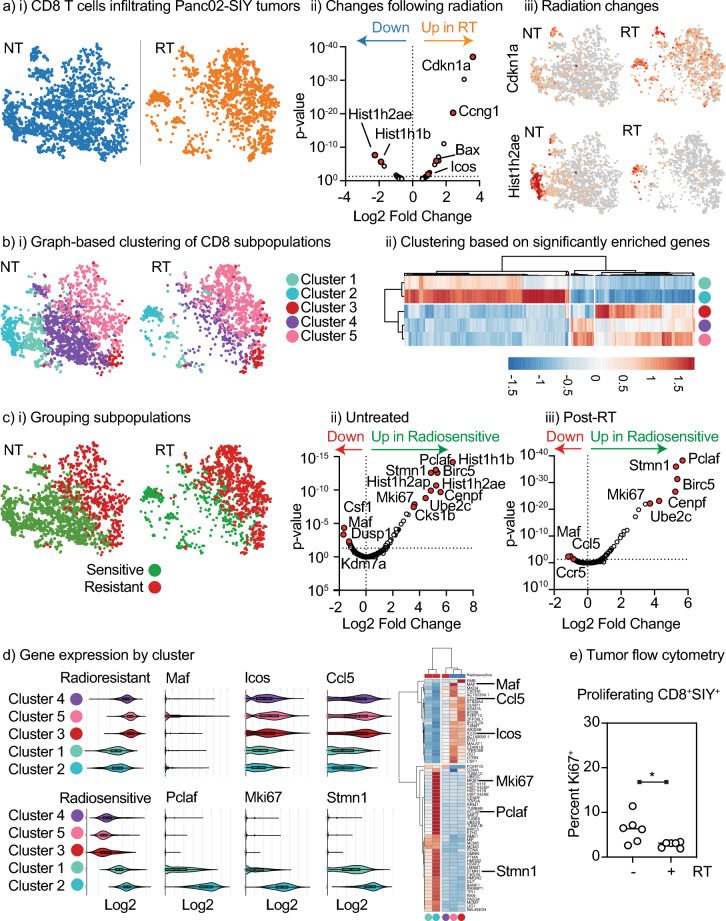
Radiation selectively eliminates proliferating CD8 T cells in the tumor resulting in an enrichment for exhausted CD8 T cells. scRNASeq of CD45^+^ cells infiltrating Panc02-SIY tumors left untreated or treated 1 day prior with 12 Gy RT was subgated for cells expressing Cd3e and Cd8a and reclustered. (**a**) (**i**) t-SNE plot shows CD8 T cells in untreated (NT) versus irradiated (RT) tumors. (**ii**) Volcano plot showing differential gene expression between NT and RT tumors, with key genes marked. (**iii**) Expression of Cdkn1a and Hist1h2ae in CD8 T cells. (**b**) (**i**) Distinct clusters from graph based clustering of CD45^+^ cells that are within the CD8 T cell population. (**ii**) Cluster plot showing differentially expressed genes that are locally different between the populations. (**c**) (**i**) Recategorizing clusters as radiosensitive or radioresistant Volcano plots showing differentially expressed genes between radiosensitive and radioresistant CD8 T cells in (**ii**) untreated and (**iii**) irradiated tumors. Key genes are marked. (**d**) Expression of a panel of genes enriched in radioresistant versus radiosensitive CD8 T cells, as a violin plot and as a cluster plot, with individual examples of each highlighted. (**e**) Panc02-SIY tumors were left untreated or treated with 12 Gy RT and harvested 24 h later. Tumors were analyzed by flow cytometry for Ki67 expression in CD8^+^ SIY-specific T cells. Key. *p < 0.05.

To understand whether tumor radiation results in loss of any specific T cell populations in the tumor, we performed an unbiased analysis of tumor infiltrating cells using single cell RNA sequencing of Panc02-SIY tumors that were untreated or harvested 1 day following radiation therapy with 12 Gy focal radiation^15^. To focus on the CD8 T cell population, we selected cells co-expressing Cd3e and Cd8a for further analysis (Supplemental Fig. 7). We compared gene expression between CD8 T cells from untreated and irradiated tumors (Fig. 4ai). Following radiation therapy, we see clear upregulation of some genes (Supplementary Table 1), notably including Cdkn1a and Ccng1 (Fig. 4aii) that are both p53-inducible genes known to be upregulated following radiation therapy^30–32^. Among the down-regulated genes are Hist1h2ae and Hist1h2ab, replication-dependent histone variants associated with proliferative cells^33,34^. Profiling expression of Cdkn1a across the CD8 T cells in the tumor, we see that Cdkn1a is broadly upregulated across all T cell populations in the tumor (Fig. 4aiii), suggesting that these cells were present at the time of radiation. By contrast, Hist1h2ae is restricted to a subpopulation of cells that is negatively impacted by radiation therapy (Fig. 4aiii). Together, these data suggest that 1 day following treatment radiation therapy caused a DNA damage response in the T cells that were in the tumor at the time of radiation and has decreased the proportion of proliferating cells. To define whether the change in proliferation is a broad impact across all T cells or a result of changes in specific T cell populations following radiation therapy, we used graph-based clustering to identify 5 major subpopulations of CD8 T cells in the tumors (clusters 1, 2, 3, 4, and 5) among other tumor infiltrating immune cells (Supplemental Fig. 7, Fig. 4bi). Following radiation therapy, CD8 T cell clusters 1, 2, and 4 are depleted while clusters 3 and 5 appear unaffected, suggesting loss of specific subpopulations. To identify distinguishing features of these subpopulations we analyzed genes differentially expressed between these subpopulations. Clusters 1 and 2 expressed significantly more genes associated with proliferation, including Mki67 and Hist1h2ae (Supplemental Table 2). Cluster 3 expressed higher levels of Itgae (CD103) and Cluster 5 higher levels of Lag3, suggesting that these represent distinct memory populations in the tumor immune environment and they appear to be less depleted by radiation. Clustering these populations based on the genes that are significantly enriched in any cluster identified that clusters 1 and 2 were clearly distinct from clusters 3, 4, and 5 (Fig. 4bii) despite cluster 4, like clusters 1 and 2, being sensitive to radiation therapy. To identify differences between the radiation sensitive and radiation resistant populations in the tumor, we grouped the T cell populations from untreated tumors and irradiated tumors into two groups: sensitive (Clusters 1, 2, 4), and resistant (Clusters 3, 5) (Fig. 4ci). In both untreated and irradiated tumors, the cells that are sensitive to radiation show increased expression of a range of genes associated with proliferation, including Pclaf, Stmn1, and Mki67 (Fig. 4cii,iii). These data suggest that populations of proliferating T cells are negatively impacted by radiation therapy. To map these genes to specific clusters, we profiled expression of all significantly regulated genes or individual genes in each cluster (Fig. 4d). Notably, clusters 1 and 2 were again clearly distinct, exhibiting higher expression of proliferative genes and lower expression of genes associated with the radioresistant population, where radioresistant populations express genes such as Icos and Ccl5. As before, Cluster 4 most closely resembled the radioresistant populations 3 and 5, indicating that despite the grouping of Cluster 4 with Clusters 1 and 2 as cells that decrease in proportion following radiation, gene expression from the proliferative Clusters 1 and 2 dominates the differential analysis following radiation therapy. For this reason, we directly compared gene expression between the radioresistant Cluster 5 to the radiation sensitive populations 1 and 2 in untreated tumors (Supplemental Fig. 8, Supplemental Tables 3 and 4). These data allow us to more clearly enrich for unique genes expressed by Cluster 5 and the analysis show that the T cells that are less sensitive to radiation therapy express a range of markers associated with exhaustion, including Lag3 and Havcr2 (Tim3), and additional immunotherapy targets Ctla4 and Icos (Supplemental Fig. 8). To validate these scRNASeq data using a different method, we performed flow cytometry of tumors at the same time point following radiation therapy and we see a loss of Ki67^+^ cells in the tumor, consistent with the scRNASeq data (Fig. 4e). These data suggest that radiation therapy is selectively eliminating the proliferating population in the tumor while having less impact on exhausted cells in the tumor. Importantly, these data agree with the impact of radiation on photoconverted cells reaching the TdLN, showing that effector cells are disproportionately negatively impacted by radiation therapy (Fig. 3).

### Impact of radiation on distant tumor immune surveillance

**Figure 5:**
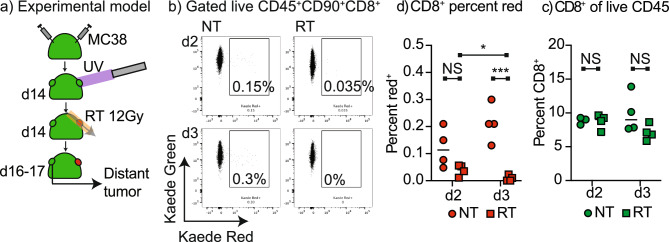
Radiation limits systemic tumor immunosurveillance. (**a**) Kaede mice were implanted simultaneously with dual MC38 tumors and at d14 one tumor was selectively photoconverted with UV light. The photoconverted tumor was left untreated or was irradiated with 12 Gy CT-guided radiation to the tumor using a SARRP. The non-photoconverted distant tumors were harvested 2 or 3 days following photoconversion. (**b**) Representative flow cytometry of CD8 T cells in the distant tumor showing the proportion that were photoconverted. (**c**) The proportion of CD8 T cells in the distant tumor and (**d**) the percent CD8 T cells that are photoconverted in the distant tumor at each time point. Key. *NS* not significant; *p < 0.05; **p < 0.01; ***p < 0.001.

To determine whether tumor radiation therapy impacted immune surveillance of distant tumors, we established MC38 tumors on both flanks of Kaede mice. Tumors were established simultaneously to minimize differences in tumor size and avoid the vaccine boost effect of delayed distant tumor challenge^29^ which may impact the pattern of lymphocyte movement. The tumor on one flank was photoconverted and randomized to no treatment or 12 Gy radiation therapy and the distant tumor was harvested 2 or 3 days later (Fig. 5a). Where the photoconverted tumor was left untreated, we saw a population of photoconverted CD8 T cells in the distant tumor on days 2 and 3 following photoconversion (Fig. 5b,c). By contrast where the photoconverted tumor was irradiated, there were significantly fewer photoconverted CD8 T cells in the distant tumor on day 2 and day 3, and in the irradiated groups there was a significant decrease in photoconverted CD8 T cells in the distant tumor between day 2 and 3 (Fig. 5b,c). Notably, there was no change in the overall number of CD8 T cells in the distant tumor on treatment, indicating that the loss of recirculation is not notable without the Kaede marker (Fig. 5d). These data demonstrate that systemic movement of T cells between tumors is impaired following radiation therapy.

### Resumption of recirculation following radiation therapy

**Figure 6:**
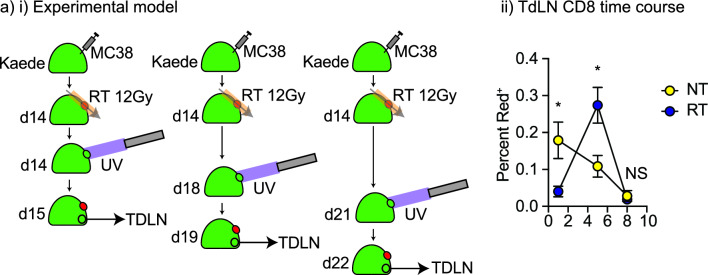
Timeline of dynamic impacts of radiation on CD8 T cell recirculation to the TdLN. (**a**) (**i**) Kaede mice were implanted with MC38 tumors and at d14 the tumor was left untreated or was irradiated with 12 Gy CT-guided radiation using a SARRP. In addition, at d14, 18, or 21 the tumor was selectively photoconverted with UV light. The TdLN were harvested 1 day following photoconversion, resulting in analysis of recirculation rates to the TdLN 1, 5, or 8 days following radiation. (**ii**) The proportion of CD8 T cells that are photoconverted in the TdLN at each time point. Key. *NS* not significant; *p < 0.05.

A range of studies have demonstrated that the proportion of CD8 T cells in irradiated tumors increase in comparison to untreated tumors 5–10 days following radiation^35–37^. To understand whether this recruitment results in a resumption in recirculation through the TdLN, we tested additional timepoints for photoconversion of the tumor. MC38 tumors were established in Kaede mice and randomized to 12 Gy RT or no treatment (Fig. 6). Photoconversion occurred either at the time of radiation or 4 or 7 days following radiation then the tumor and TdLN were harvested 1 day later (d1, d5, or d8 respectively). At d5 we observed that total T cell numbers in the irradiated tumor were fully restored compared to untreated tumors (Supplemental Fig. 9) and that the number of photoconverted CD8 T cells in the TdLN of irradiated tumors were significantly increased compared to untreated tumors (Fig. 6). At day 8, the rate of CD8 T cell movement to the TdLN was not significantly different between untreated and irradiated tumors suggesting that recirculation has normalized by this time point (Fig. 6). These data illustrate a timeline of tumor refilling post-radiation that includes a high rate of CD8 T cell movement through the TdLN that may play a key role in antigen-guided T cell control of residual cancer cells and systemic tumor immune surveillance following radiation.

## Discussion

This study demonstrates that radiation transiently impairs T cell recirculation due to direct cytotoxicity to radiosensitive T cells in the treatment field. We observe that tumors that are highly infiltrated with T cells also have higher rates of recirculation to the TdLN, dynamically linking infiltration to immunosurveillance^3^. Importantly, we demonstrate that radiation of the tumor also transiently negatively impacts T cell movement beyond the draining lymph node to distant tumors and lymph nodes.

The fact that radiation is cytotoxic to T cells generates a paradox given that tumor-specific T cells are enriched in the treatment field and therefore preferentially depleted by radiotherapy, yet tumor specific T cells are required for the full efficacy of radiation therapy in preclinical models (reviewed in^1^). Prior studies have demonstrated that T cells in the tumor are less sensitive to radiation than T cells in the peripheral circulation following total body radiation^38^, but despite this relative radioresistance all of the major CD8 T cell populations in the tumor are negatively affected by the higher doses radiation doses used in hypofractionated radiation therapy^28,38^. Our data demonstrate that the loss of T cells following radiation also impacts the TdLN, with T cell recirculation from the tumor to the TdLN transiently lost as a result of loss of T cells in the treatment field.

While the Kaede photoconversion system allows us to study the movement of cells in novel ways, there are limitations to photoconversion. UV light has a limited depth of penetration and thus photoconversion at depth is incomplete^17^. For this reason we have focused on the positively converted cells, the Kaede Red^+^ cells, since we cannot definitively determine the origin of the unconverted cells. In the photoconverted tumors we observe up to 80% of cells converted immediately following UV exposure^15^, and a decline over time. 1 day following photoconversion we commonly observe 30–60% of cells in the tumor remain photoconverted, but over time this will be impacted by cell replacement as well as loss of signal. In addition, it is possible that sub-regions of the tumor are differentially photoconverted, which may impact results. While we have not identified any cell populations that are differentially represented in the photoconverted versus unconverted populations in the tumor, further work is needed to determine whether cell movement is dominated by cells originating in tumor sub-regions. In addition, these studies highlight that a very small number of cells are moving at any one time. The analysis of this population risks spurious results due to the potential for extreme variation in any small subpopulation. However, such variation would be expected to be random, so in addition to our replication of experiments to address this we replicated the data in four different tumor models with consistent outcomes. These data suggest that these represent a genuine migrating cell population with consistent phenotype and response to treatment. One important point is that these small numbers can have outsized importance. For example, in our prior studies we saw similarly small numbers of DC migrating to the TdLN but when these were blocked from migrating, we lost T cell control of tumors^15^. Understanding the importance of cell movement on immune control may necessitate studying small numbers of cells hidden among larger populations.

Radiation may impact cell movement from tumors independent of direct T cell cytotoxicity via its effects on the lympho-vasculature. Radiation has been shown to decrease the presence of lymphovascular structures in normal tissues and this loss follows the progressive timecourse of fibrotic development. Thus, marked changes in lympho-vasculature and lymphatic clearance take many weeks to emerge^39,40^. However, while loss of lymphovascular cells can be slow, there may be early effects on fluid flow observable within 1d of radiation to normal tissue at 20 Gy and 40 Gy single doses^41^. This is consistent with rapid loss of lymphovascular contractility in normal tissue following a single 10 Gy dose of radiation, resulting in changes in fluid flow^42^. While these events may be taking place in the tumor, we did not observe a significant change in DC migration to the TdLN following radiation, suggesting that cellular transit is not impaired in the timeline of the study. In addition, we see loss of T migration to the TdLN at fractionated 2 Gy × 3 and 6 Gy × 1, which are generally below the threshold to observe vascular impacts of radiation. Nevertheless, the dynamic of cell and fluid communication between the tumor and the TdLN may have additional impacts beyond the effect of radiation on cell death in the treatment field.

In tumors, CD8 T cells can exhibit distinct features associated with exhaustion. As would be expected, the CD8 T cells that are specific for tumor-associated antigens are more likely to exhibit these exhaustion phenotypes^43^. There is ongoing debate whether exhausted cells can recover to again participate in the tumor immune response, or whether these cells are terminally exhausted^44–48^. Our RNASeq data demonstrate a selective loss of proliferating T cells and the T cells that remain following radiation are enriched for expression of genes such as Ccl5 and Csf1 which have been associated with tumor promotion by T cells^49^ and dysfunctional T cells in human tumors^46^ respectively. The more resistant cells have the highest expression of the exhaustion markers Havcr2 and Lag3, which is consistent with these being terminally exhausted CD8 T cells^50^. This expression of immunotherapy targets such as Havcr2, Lag3, and also Icos in the T cells that remain following radiation may explain their potential usefulness as targets in combination with radiation therapy to help eliminate residual disease^51–54^. It remains possible that the alterations in the T cell populations in the tumor environment following radiation are not the result of death of specific populations, but phenotypic shifting. In this way proliferative cells may halt proliferation and upregulate activation and exhaustion markers, resulting in an apparent loss. However, we would anticipate that T cells that are not killed by radiation would be halted at the G2/M checkpoint over the timeline of this study^55^, where expression of proliferation-related genes such as Mki67 would remain^56^. Thus, the loss of Mki67 following RT is more consistent with cytotoxicity than G2/M arrest. Phenotypic shifting may be more relevant for the less clearly defined non-proliferating populations that are phenotypically close to the exhausted T cells. In these cells, rapid upregulation of exhaustion markers may mean that the cells shift into an apparently exhausted phenotype such that their original phenotype is lost following radiation. This may explain the apparent radiosensitivity of cluster 4 better than differential sensitivity to radiation. Further studies are needed to distinguish these mechanisms. The loss of proliferative cells may have unknown negative effects on tumor control. Recently a progenitor exhausted, or stem-like population has been defined that may be more responsive to checkpoint inhibition^44,45^. This population appears to recirculate to a greater degree^16^ and is also more radiosensitive^10^. Our data demonstrates that when there is more than one tumor in mice T cells recirculate from one tumor to another, which may contribute to systemic immune surveillance. Following radiation, cells from the treated tumor are no longer available to enter the other tumor and the increased recruitment of T cells to the refilling tumor may negatively impact systemic immune surveillance. In this setting the treated tumor environment and the associated TdLN may be critical sites to propagate immunity to distant tumors, or alternatively suppress systemic immunity, depending on the immune consequence of radiation. For patients with widely metastatic tumors, recent data suggests that passage of T cells through liver metastases impairs systemic immunity, such that radiation-mediated elimination of the liver metastasis improves responses to immunotherapy^57^. These data demonstrate that the movement of T cells through multiple sites is an underappreciated mechanism explaining treatment success and failure.

These data also demonstrate that repopulation of the tumor following radiation occurs rapidly and is associated with a high rate of movement of cells through the tumor to the TdLN. This high rate of movement may be linked to the relatively low number of tumor-specific T cells that are present in peripheral sites: for example, in the Panc02-SIY model less than 0.5% of T cells in the spleen or NdLN are tumor-specific^29^ and the number in the peripheral blood rapidly drops below detectable thresholds as tumors progress^28^. Thus, early recruitment is dominated by T cells that are not tumor specific and will therefore meet few retention signals in the tumor environment. To provide additional tumor-specific T cells the timeline of T cell expansion in the TdLN following radiation likely depends on RT-mediated adjuvant release, which can take 2–5 days following RT^58,59^, and movement of matured dendritic cells, which becomes detectible 2–3 days following RT^15^. Tumor-specific T cell expansions in the TdLN of irradiated tumors has been described 7 days following radiation^37^. Yet, irradiation of the TdLN at the same time as tumor irradiation impairs tumor control by T cells^11^, which suggests that the critical T cells are already in the TdLN at the time of radiation. These data suggest that the dynamics and timeline of T cell control of tumors following radiation requires further understanding, which may be critical to optimize adaptive immune control of residual disease.

## Methods

### Animals and cell lines

Animal protocols were approved by and performed according to the requirements of the Earle A. Chiles Research Institute (EACRI) Institutional Animal Care and Use Committee (Animal Welfare Assurance No. D16-00526). Experiments were performed according to ARRIVE guidelines. Blinding was not used during experimental procedures. For tissue harvest the mice were euthanized by CO_2_ inhalation followed by cervical dislocation. Kaede transgenic mice were kindly provided by Dr. Amanda Lund at Oregon Health and Science University as previously described^15^. Experiments were performed with 8–12-week-old mice with 4–8 female mice per group. The Panc02-SIY pancreatic adenocarcinoma line expressing the model antigen SIY was kindly provided by Dr. Ralph Weichselbaum at the University of Chicago. The MC38 colorectal carcinoma line was obtained from Dr. Kristina Young at EACRI. The Moc1 and Moc2 oral squamous cell carcinoma lines were kindly provided by Dr. Ravindra Uppaluri at the Dana Faber Cancer Institute. Pathogen and mycoplasma contamination testing were performed on all cell lines using the IMPACT II Mouse PCR Profiling from IDEXX BioAnalytics.

### Tumor treatments

Tumors were implanted subcutaneously into the right flank as follows; 2 × 10^5^ MC38, 5 × 10^6^ Panc02-SIY, 1 × 10^6^ Moc1, and 1 × 10^5^ Moc2. When tumors were approximately 5 mm in average diameter at approximately 14 days following implantation, mice were randomized to receive treatment with CT-guided radiation using the Small Animal Radiation Research Platform (SARRP) from XStrahl. Dosimetry was performed using Murislice software from XStrahl. The SARRP delivered a single dose of 12 Gy to an isocenter within the tumor using a 10 mm × 10 mm collimator and a 45° beam angle to minimize dose delivery to normal tissues. For photoconversion experiments using the Kaede mice, tumors were converted as previously described^15^. Briefly, tumors were implanted in animals with shaved skin, and for photoconversion animals were completely covered in aluminum foil except for the tumors which were exposed to 405 nm LED light source using a collimator for 5 min (Prizmatix). Where radiation therapy followed Kaede conversion, the UV conversion in Kaede mice was performed immediately prior to radiation therapy, with at most a 1 h lapse between conversion and radiation treatment.

### Tissue processing

Following dissection, tumors were weighed and minced into small fragments, then transferred into C tubes from Miltenyi Biotec containing enzyme digest mix with 250 U/mL collagenase IV (Worthington Biochemical, #LS004188), 30 U/mL DNase I (Millipore-Sigma, #4536282001), 5 mM CaCl_2_, 5% heat inactivated FBS and HBSS. Tissue was dissociated using a GentleMACS tissue dissociator from Miltenyi Biotech. This was followed by incubation at 37 °C for 30 min with agitation. For the lymph nodes, capsules were cut open and incubated with enzymatic mix described above at 37 °C for 15 min with agitation. Enzyme mix containing lymph nodes was then vigorously pipet mixed and incubated at 37 °C for an additional 15 min. Enzymatic reactions for both the tumor and lymph nodes were quenched using ice cold RPMI containing 10% FBS and 2 mM EDTA. Single cell suspensions were then filtered through 100 µm (tumor) or 40 µm (lymph nodes) nylon cell strainers to remove macroscopic debris. Cells were washed and the number and viability of cells was determined using a Guava (EMD Millipore).

### Flow cytometry

For surface staining, 2 × 10^6^ cells were stained with Zombie Aqua Viability Dye from BioLegend (#423102) in PBS for 10 min on ice, then Fc receptors were blocked with α-CD16/CD32 antibodies from BD Biosciences (2.4G2) for an additional 10 min. After centrifugation, the supernatant was removed and cell were stained with a surface antibody cocktail containing in FACS buffer (PBS, 2 mM EDTA, 2% FBS) and Brilliant Stain Buffer Plus from BD Biosciences (#566385) for 20 min on ice. After surface staining, cells were washed in FACS buffer and fixed for 20 min on ice with Fixation/Permeabilization Buffer from BD Biosciences (#554722). For intracellular staining of Kaede^+^ cells, we used a modified version of the protocol described by Li et al.^16^. Cells were surface stained as above, then the surface stains were washed out. Cells were treated with Cytofix/Cytoperm from BD Biosciences (# 554722) on ice in the dark for 30 min then washed with eBioscience Foxp3/Transcription Factor Staining Buffer Set (# 00-5523-00) and intracellularly stained with antibodies in eBioscience Foxp3/Transcription Factor Staining Buffer on ice in the dark for 30 min. Cells were washed and resuspended in FACS buffer for flow cytometry. Surface stains include the following antibodies from BioLegend; CD90.2-A700 (30-H12), CD8a-BV650 (53-6.7), CD4-APC-Cy7 (RM4-5), CD62L-BV605 (MEL-14), CD44-BV711 (IM7), F4/80-PerCP/Cy5.5 (BM8), CD11c-PE/Cy7 (N418), CD19-A700 (6D5), MHC-II-BV421 (M5/114.14.2), CD11b-BV605 (M1/70), and Ly-6C-BV711 (HK1.4). CD3-Alexa700 (17A2), CD69-APC (H1.2F3), CD25-BV421 (PC61), CD103-APC (2E9), and CD24-APC e780 (M1/69) were obtained from Thermo Fisher Scientific. CD45-BV786 (30-F11) was purchased from BD Biosciences. Intracellular stains include TCF7/TCF1-Alexa647 (S33-966) from BD Biosciences, and Ki67-BV421 (16A8) from BioLegend. All samples were resuspended in FACS buffer and acquired on a BD Fortessa flow cytometer. Data were analyzed using FlowJo software from Tree Star, v10.8.

### Single cell RNA sequencing

The datasets used for single cell RNA Sequence analysis was previously published^15^ and have been deposited in NCBI's Gene Expression Omnibus^60^, accessible through GEO Series accession number GSE201026 (https://www.ncbi.nlm.nih.gov/geo/query/acc.cgi?acc=GSE201026). Data were analyzed with the Loupe Browser from 10X Genomics (v5.0).

### Statistics

Data were analyzed and graphed using Prism from GraphPad Software (v9.0). Individual data sets were compared using Student’s *t*-test and analysis across multiple groups was performed using one-way ANOVA with individual groups assessed using Tukey’s comparison.

### Supplementary Information

Supplementary Information.

Supplementary Table 1.

Supplementary Table 2.

Supplementary Table 3.

Supplementary Table 4.

## Data Availability

All data is present in the manuscript, supplemental figures, and attached data.
