# Supplementary material for: Fluorescence tracking demonstrates T cell recirculation is transiently impaired by radiation therapy to the tumor
Source: Sci Rep. 2024 May 24;14:11909. doi: 10.1038/s41598-024-62871-w (PMC11126658; doi:10.1038/s41598-024-62871-w)
Supplement: Supplementary file 1 — Supplementary Information. [file 41598_2024_62871_MOESM1_ESM.pdf]

Fluorescence tracking demonstrates T cell recirculation is transiently impaired by radiation therapy to the tumor.

Gwen Kramer, Tiffany Blair, Shelly Bambina, Aanchal Preet Kaur, Alejandro Alice, Jason Baird, David Friedman, Alexa K. Dowdell, Michio Tomura, Clemens Grassberger, Brian D. Piening, Marka R Crittenden, Michael J Gough

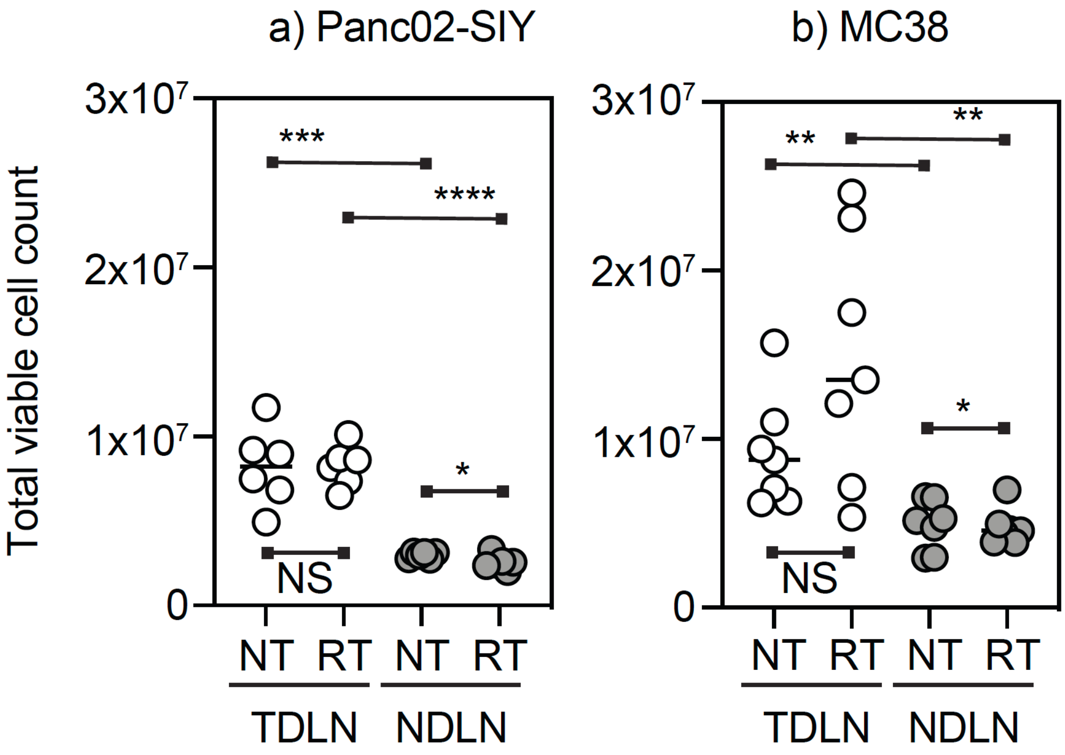

Supplemental Figure 1. Total cellularity of TdLN and NdLN

Total number of viable cells in the TDLN and NDLN of i) Panc02-SIY tumor bearing mice and ii) MC38 tumor bearing mice.Each symbol represents one animal. Key: NS = not significant; *p<0.05; ** p<0.01; *** p<0.001; **** p<0.0001

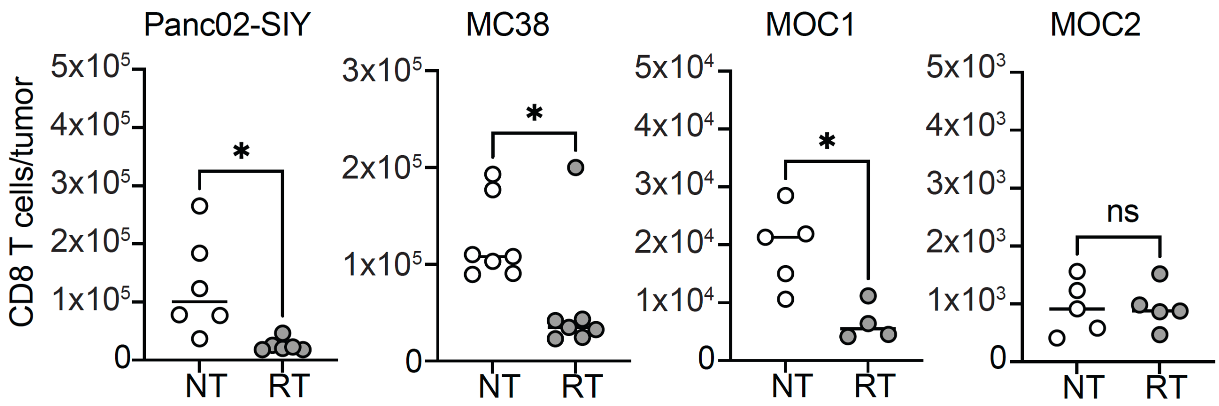

Supplemental Figure 2. CD8 T cells infiltration of tumors is decreased following RT.

Absolute number of viable CD8 T cells in the tumor of i) Panc02-SIY, ii) MC38, iii) Moc1, and iv) Moc2 tumors left untreated or 3d following 12Gy RT. Each symbol represents one animal. Key: ns = not significant; *p<0.05.

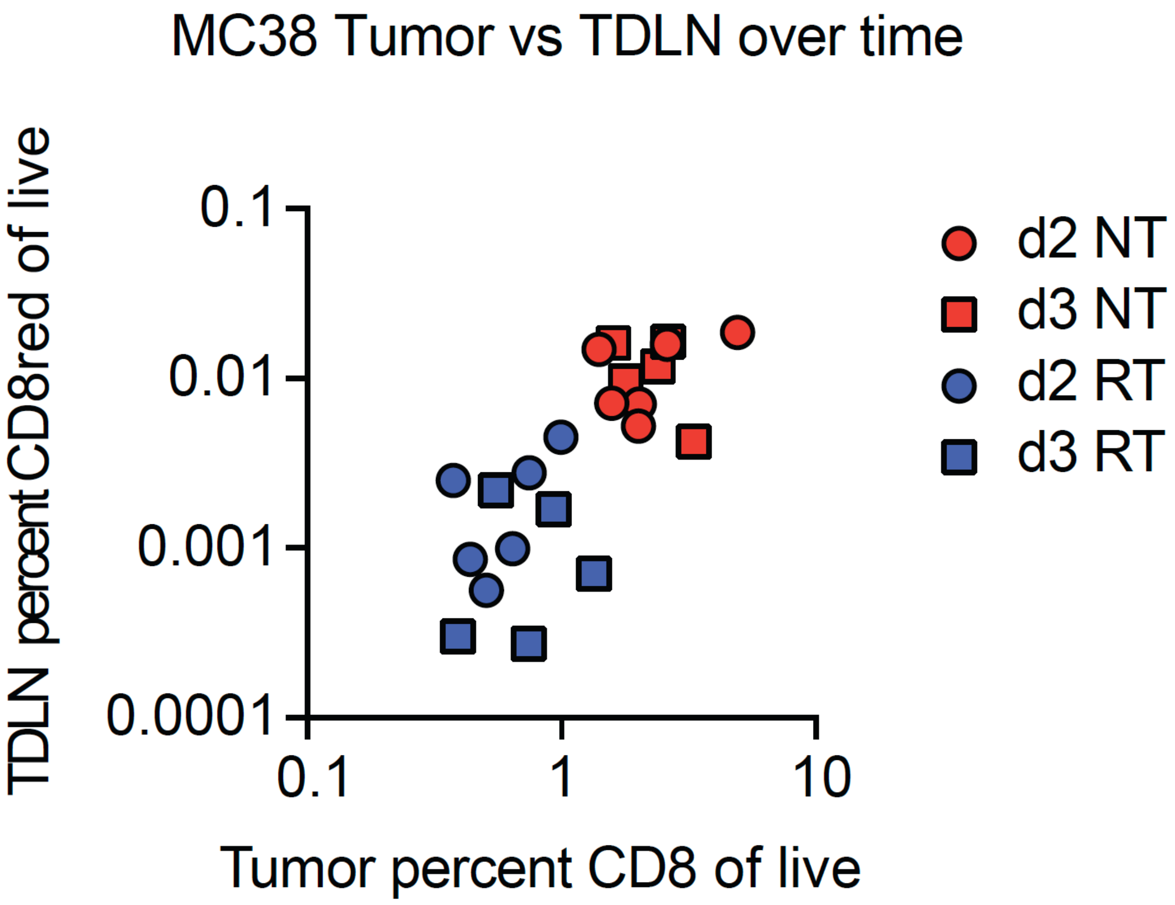

Supplemental Figure 3. Coordinate decrease in CD8 T cells in the tumor and recirculating to the TdLN is sustained over time.
Analysis of mice bearing MC38 tumors left untreated or treated with 12Gy RT 2 and 3 days following RT. Graph shows the percent CD8 T cells in the tumor versus percent photoconverted (Red+) CD8 in the TdLN. Red symbols NT, blue symbols RT. Circles d2, squares d3. Each symbol represents one animal.

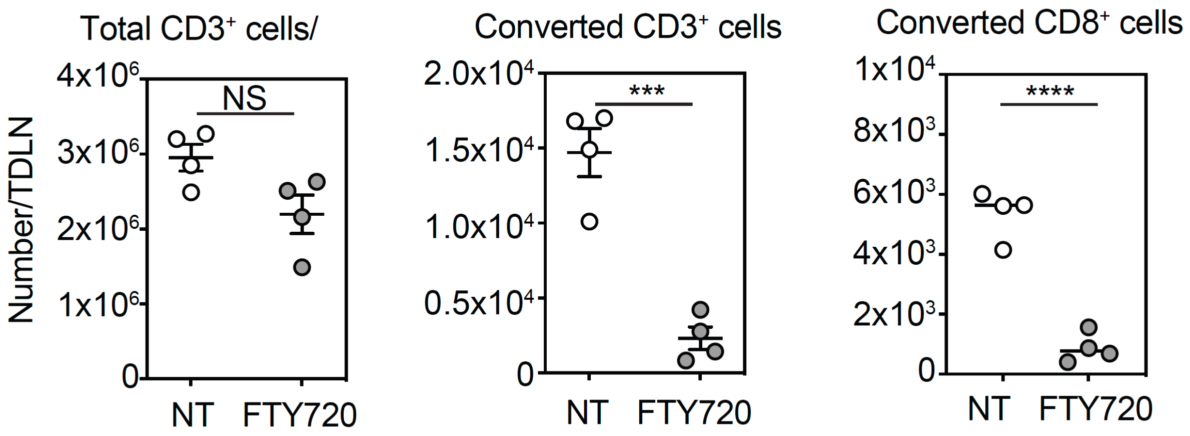

Supplemental Figure 4. Recirculation of T cells to the TdLN depends on S1P-mediated exit from the tumor.
Analysis of mice bearing MC38 tumors left untreated or treated with FTY720 and photoconverted 1 day prior to harvest. Graph shows the absolute number of CD3+ cells, converted CD3+ cells and converted CD8+ T cells in the TdLN. Each symbol represents one animal. Key: NS = not significant; *** p<0.001; **** p<0.0001

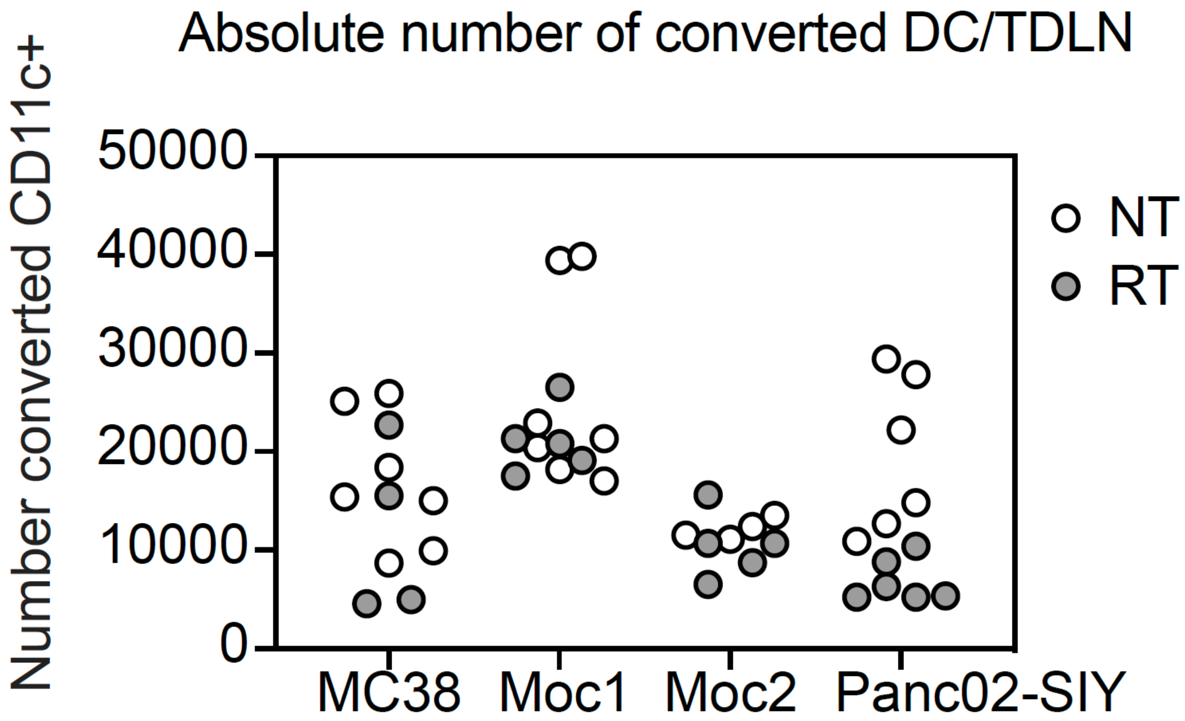

Supplemental Figure 5. Recirculation of DC to the TdLN
Analysis of TdLN of mice left untreated or treated with 12Gy RT. Graph shows the absolute number of photoconverted CD11c+ cells in the TdLN. Each symbol represents one animal.

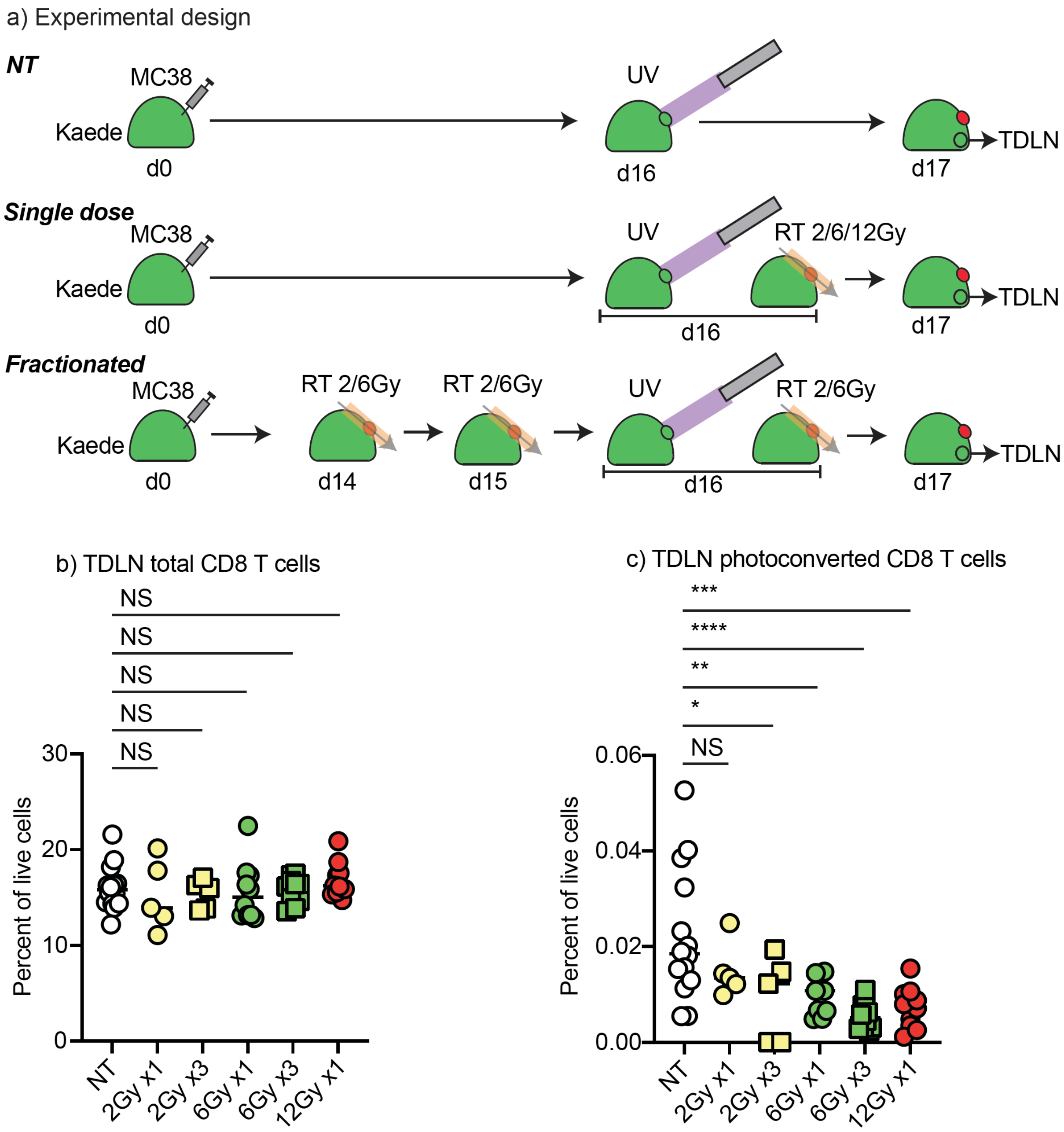

Supplemental Figure 6. Dose and fractionation impact on recirculation
A) Analysis of TdLN of mice bearing MC38 tumors left untreated or treated with RT in a range of dose and fractionation schemes. Graph shows the B) the proportion of CD8 T cells in the TdLN or C) the proportion of photoconverted CD8 T cells in the TdLN. Each symbol represents one animal. Key: NS = not significant; *p<0.05; ** p<0.01; *** p<0.001; **** p<0.0001

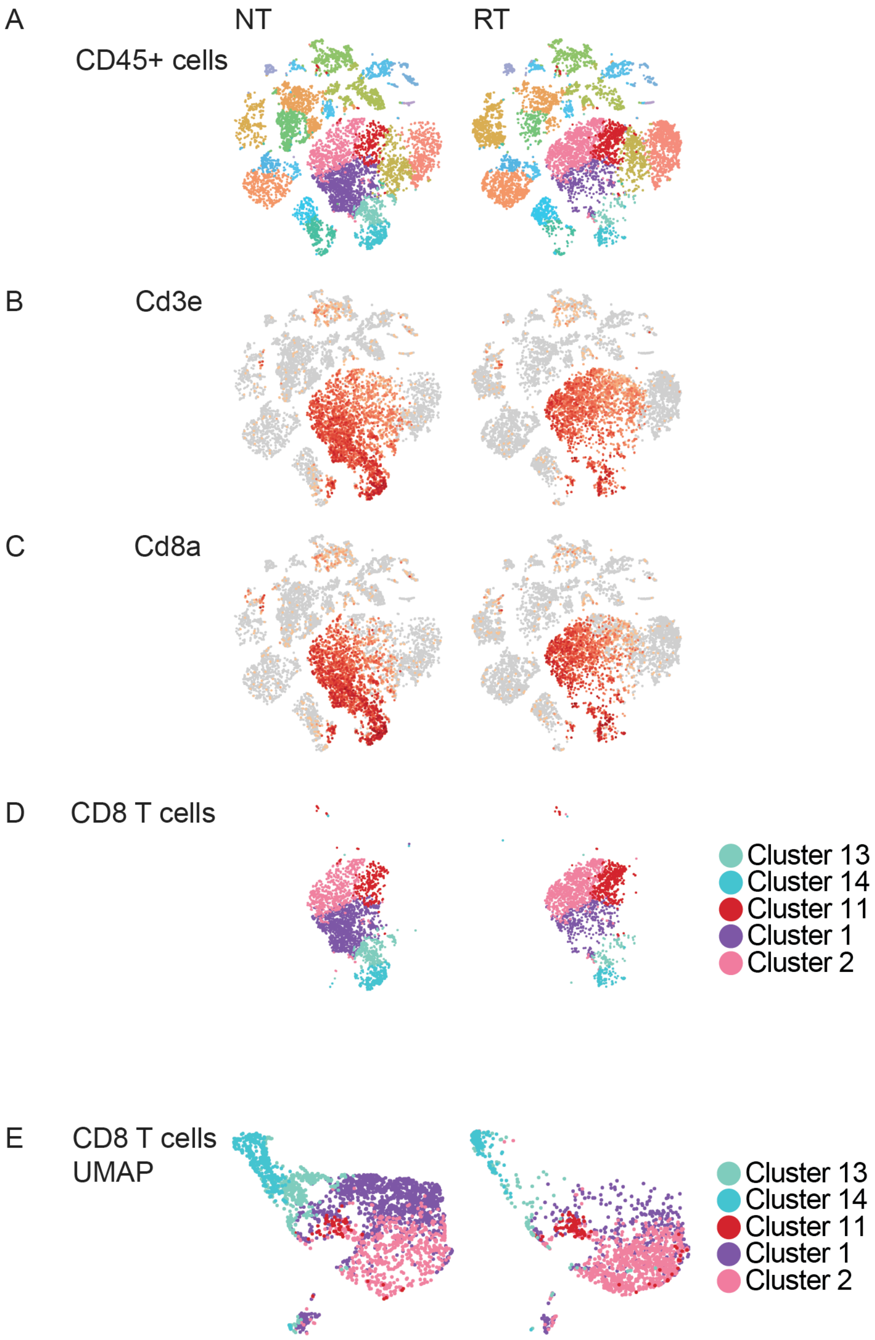

Supplemental Figure 7. Selection of CD8 T cells in scRNASeq for analysis
A) t-SNE plot of CD45+ cells in untreated versus RT Panc02-SIY tumors 1 day following 12Gy focal RT, with unbiased graph-based clustering of infiltrating cell types separated by treatment. Subgraphs show B) Cd3e and C) Cd8a expression to identify $CD8^+$ T cells, and D) the 5 major clusters that express Cd3e and CD8a. E) UMAP plot of D) showing $CD3e^+CD8a^+$ cells, separated by treatment.

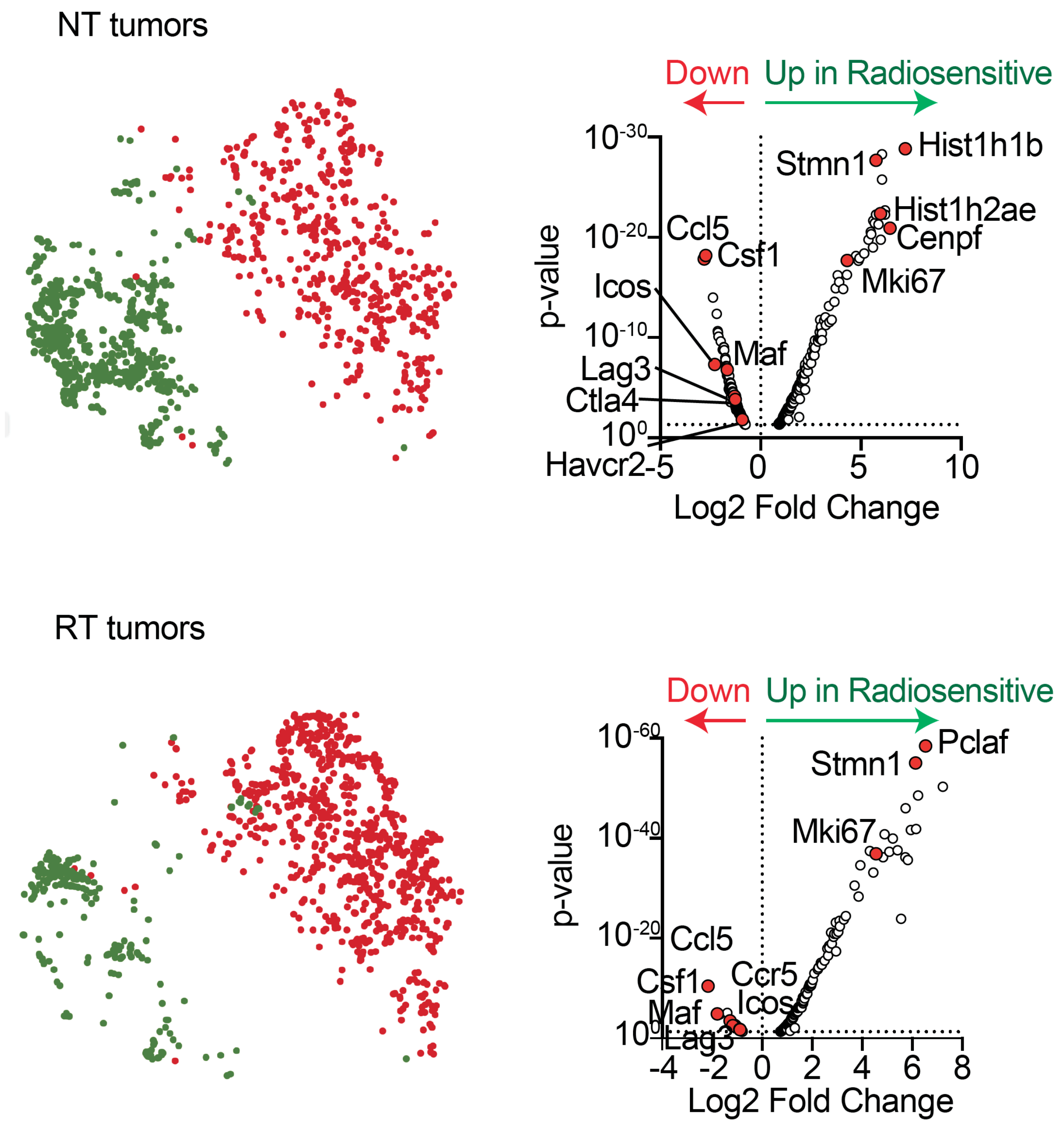

Supplemental Figure 8. Comparison of radioresistant versus radiosensitive CD8 T cells.

A) t-SNE plot of radioresistant cluster 2 (red) and radiosensitive clusters 13 and 14 (combined in green) in tumors that are untreated (NT) top and 12Gy irradiated (RT) bottom. Volcano plots show genes that different between radiosensitive and radioresistant T cells in NT and RT tumors, with key genes highlighted.

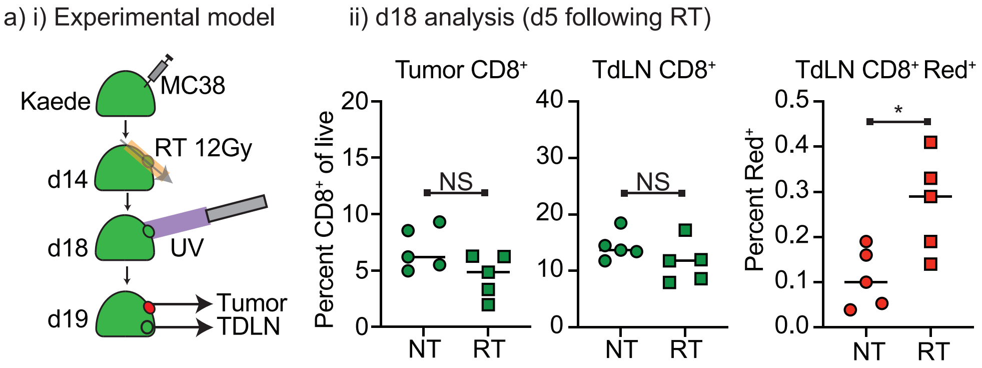

Supplemental Figure 9. Comparison of radioresistant versus radiosensitive CD8 T cells.

A) i) Kaede mice were implanted with MC38 tumors and at d14 the tumor was left untreated or was irradiated with 12Gy CT-guided radiation using a SARRP. At 18 the tumor was selectively photoconverted with UV light. The TdLN was harvested 1 day following photoconversion, resulting in analysis of recirculation rates to the TdLN 5 days

following radiation. ii) The proportion of CD8 T cells that are photoconverted in the TdLN at d18 (5 days post-RT). Key. NS=not significant; *=$p<0.05$.

Supplemental Tables

Supplemental Table 1. Genes differentially expressed in Cd3e+ CD8a+ T cells d1 following radiation therapy

Supplemental Table 2. Genes differentially expressed in 5 graph-based clusters of cells identified in Cd3e+ CD8a+ T cells in untreated tumors

Supplemental Table 3. Genes differentially expressed between radiosensitive and radioresistant clusters in untreated tumors.

Supplemental Table 4. Genes differentially expressed between radiosensitive and radioresistant clusters in irradiated tumors.
